# Role of titanium and organic precursors in molecular layer deposition of “titanicone” hybrid materials

**DOI:** 10.3762/bjnano.13.103

**Published:** 2022-11-02

**Authors:** Arbresha Muriqi, Michael Nolan

**Affiliations:** 1 Tyndall National Institute, University College Cork, Lee Maltings, T12 R5CP Cork, Ireland

**Keywords:** density functional theory (DFT) studies, double reactions, surface chemistry, titanicone

## Abstract

The development of hybrid inorganic–organic films with well-controlled properties is important for many applications. Molecular layer deposition (MLD) allows the deposition of these hybrid films using sequential, self-limiting reactions, similar to atomic layer deposition (ALD). In this paper, we use first principles density functional theory (DFT) to investigate the growth mechanism of titanium-containing hybrid organic–inorganic MLD films, known as “titanicones”. We investigate in detail the chemistry between the most common Ti precursors, namely titanium tetrachloride (TiCl_4_) and tetrakis(dimethylamido)titanium (Ti(DMA)_4_), and ethylene glycol (EG) and glycerol (GL) as the organic precursors. We analyse the impact of the substrate on the initial MLD reactions in titanicone film growth using three different surface models: anatase TiO_2_, rutile TiO_2_ and Al_2_O_3_. Calculated energetics show that while TiCl_4_ is reactive towards the anatase and rutile TiO_2_ surfaces, it is not reactive towards the Al_2_O_3_ surface. Ti(DMA)_4_ is reactive towards all surfaces. This is attributed to the stronger Ti–Cl bonds in TiCl_4_ compared to Ti–N bonds in Ti(DMA)_4_. Ti(DMA)_4_ also shows high reactivity to the organics compared to TiCl_4_. Double reactions of EG and GL with the TiCl_3_ species from TiCl_4_ and TiDMA species from Ti(DMA)_4_ are also explored to better understand the origin of the different thicknesses of EG–titanicone and GL–titanicone films observed in experimental work. We find that EG and GL coupled with TiCl_4_ can orient in a flat lying configuration on anatase while on rutile, the preferred orientation is upright. When combined with Ti(DMA)_4_, EG and GL prefer the flat lying configuration on all surfaces. This work shows that the choice of the surface and the metallic precursor has a major impact on the behaviour of organic species. DFT findings provide motivation to develop a low temperature rutile TiO_2_/titanicone film suggesting that the desired film growth could be achieved.

## Introduction

Molecular layer deposition (MLD), a thin film deposition technique, has attracted significant attention in recent years as a suitable approach for the deposition of organic–inorganic hybrid films for applications in several technological application areas, including packaging/encapsulation, electronics, batteries and biomedical applications [1–4]. MLD is very similar to the widely used atomic layer deposition (ALD) technique, which involves the fabrication of inorganic films used extensively in photovoltaics, (nano)electronics, energy storage and catalysis [5–8]. Similarly to ALD, MLD is based on sequential self-limiting reactions of readily vaporized inorganic precursors but the second reactant is a highly volatile organic species. Thus, in contrast to ALD, in MLD the deposition chemistry can be extended by including organic precursors, leading to the deposition of hybrid organic–inorganic MLD films [3,9]. Two organic precursors can also be employed to deposit pure organic MLD films, such as polymers [10–12]. Similar to ALD, MLD enables the deposition of conformal and smooth films with a controlled thickness at Angstrom level [3,9].

The advantages offered by hybrid organic–inorganic MLD films are that they are ultrathin films with high flexibility, have tunable properties and excellent mechanical and electronic properties resulting from the combination of the individual properties of the organic and inorganic components that are incorporated into the film [1–2,13–15].

A special class of hybrid organic–inorganic films are so-called metalcones, which are fabricated via MLD using organometallic precursors and organic alcohols. These films are described as O–M–O–(CH*_x_*)*_y_*–O–M–O. Metalcones are known to be flexible in nature due to the flexible organic backbones present in their architectures and with excellent mechanical properties at the atomic and molecular level arising from the organometallic precursors [16–17]. The first metalcone films were Al based and were known as “alucones” [18–20]. Soon after, other metalcone groups were developed, such as the Ti, Zn, Hf, Mg and V based metalcones known as “titanicones”, “zincones” [21–22], “hafnicones” [23], “magnesicones” [24] and “vanadicones” [25], respectively. While other organic backbones, e.g., aromatic rings, have been used, the field tends to use the term “metalcone” as a general description for these hybrid materials.

One of the most extensively researched metalcones are titanicones. Titanicones are deposited by coupling a titanium inorganic precursor, such as titanium tetrachloride (TiCl_4_), with organic alcohols such as ethylene glycol (EG) [26–31], glycerol (GL) [29–31] or fumaric acid (FC) [32]. Tetrakis(dimethylamino)titanium (Ti(N(CH_3_)_2_)_4_), henceforth denoted Ti(DMA)_4_) was also successfully employed as Ti source and combined with EG and GL to deposit titanicone films [33].

In ref [30] titanicone films were deposited using TiCl_4_ as metal source and EG or GL as organic precursors. TiCl_4_–EG films and TiCl_4_–GL films were deposited on Si(100) wafers. XRR analysis found a growth per cycle (GPC) of ≈4.6 Å/cycle for TiCl_4_–EG films at 90 °C which decreases to 1.5 Å/cycle at 135 °C. It was assumed that this drop could be related to the desorption of unreacted TiCl_4_ species at 135 °C. In addition, it was proposed that the reduction in growth rate could also be caused by the desorption of Ti(–O(CH_2_)_2_O–)_2_ species or double reactions of the organic precursor in which the molecule lies flat with both termini binding to Ti sites.

The thickness and thickness reduction with temperature increment observed for TiCl_4_–EG films was similar to the thickness and thickness reduction observed for alucone films grown using trimethylaluminium (TMA) and EG (4.0 Å/cycle at 85 °C to 0.4 Å/cycle at 175 °C) [18] and zincone films grown using diethylzinc (DEZ) and EG (4.0 Å/cycle at 90 °C to 0.25 Å/cycle at 170 °C) [21].

For TiCl_4_–GL films the growth rate was 2.8 Å/cycle at 130 °C and it decreased to 2.1 Å/cycle at 210 °C. The thickness of TiCl_4_–GL films was similar to the thickness of alucone films grown using TMA and GL (2.5 Å/cycle) [34]. Similar growth rates of TiCl_4_–EG and TiCl_4_–GL films were achieved also in ref. [31] (TiCl_4_–EG: 4.5 Å/cycle at 115 °C; TiCl_4_–GL: 2.2 Å/cycle at 150 °C).

Compared to EG, GL has an extra OH group that increases the bridging between the polymer chains in the film and this also improves the stability of the film. Nanoindentation experiments [30] showed also that GL-based titanicones have much higher elastic modulus and higher hardness compared to EG-based titanicones. Annealing experiments indicated a higher thermal stability for GL-based titanicones as well [30].

Ti(DMA)_4_ is another Ti-based inorganic precursor that was investigated as an alternative to TiCl_4_ as the titanium precursor. Ti(DMA)_4_ was combined with EG and GL to deposit titanicone films in a temperature range from 80 °C to 160 °C on an Si substrate with 100 nm thermal oxide. In situ ellipsometry revealed for EG-based titanicones that the growth initiates but terminates after only 5 to 10 cycles, while for GL-based titanicones the growth proceeds and films have a GPC of 0.9 Å/cycle to 0.2 Å/cycle as the temperature increases. This is due to the double reaction phenomenon, which for EG removes both the reactive OH groups required to couple with the Ti precursor, while even with a double reaction, GL has a third OH group available to couple to the Ti(DMA)_4_ precursor in the next cycle [33]. Ti(DMA)_4_ inorganic precursor is well known and widely used in ALD for the deposition of TiO_2_ and TiN films by thermal and plasma-enhanced processes [35–36].

As described above, TiCl_4_ and Ti(DMA)_4_ were successfully employed in MLD of titanicone films [30,33]. While TiCl_4_ is a small halide molecule that has Ti–Cl bonds, Ti(DMA)_4_ is a bulkier molecule with Ti–N bonds. Bond dissociation energy values for the breakage of the Ti–Cl and Ti–N bonds serve as a measure of the stability of these precursors and these are 494 kJ/mol and 464 kJ/mol for Ti–Cl and Ti–N, respectively. Bond dissociation energies indicate that a lower energy is needed to break the Ti–N bond compared to the Ti–Cl bond and hence a higher reactivity for the Ti(DMA)_4_ precursor towards the surface and towards the co-reactant is expected.

In addition, due to the bulky DMA ligands of Ti(DMA)_4_, the “reservior” effect, which is very common for small molecules such as TiCl_4_ [30], DEZ [21] and TMA [37] and which leads to uncontrolled growth is avoided. Moreover, the ligand elimination reactions of the Ti(DMA)_4_ process yield H–N(CH_3_)_2_ by-products. In contrast this is non-corrosive compared to HCl by-products released during the TiCl_4_ process. For TiCl_4_ this is considered a significant drawback [38].

Fumaric acid (FC) is another alcohol organic precursor that was used to deposit titanicone films using TiCl_4_ on an Si substrate in a temperature range of 180 °C to 350 °C. A temperature-dependent growth characteristic was observed with the growth rate decreasing from 1.10 Å/cycle at 180 °C to 0.49 Å/cycle at 300 °C. This reduction was attributed to the increased thermal motion and desorption of molecules on the growth surface. FTIR spectra indicated that the hybrid film shows a stable bridging bonding mode between Ti and the acid group at temperatures under 200 °C and a high bridging/bidentate mixed bonding mode at temperatures over 250 °C and 300 °C [32].

Many studies show that the desired properties and the target thickness of a metalcone MLD film are not actually achieved. To help understand this, first principles density functional theory (DFT) calculations have been employed to explore the reaction mechanisms, energetics and the role of the organic precursors on the growth of hybrid films [24,39–41]. DFT studies show that the aliphatic diol precursors, namely EG and GL, when combined with TMA, undergo double reactions, binding with the surface fragments through the two terminal OH groups, and this phenomenon reduces the number of active sites and terminates the surface growth [39].

However, DFT results also show that this scheme depends on the metal precursor and the surface as the same aliphatic precursors behave differently when combined with Mg(Cp)_2_; EG still prefers to orient in a flat configuration and react twice with the surface, while GL prefers to lie in an upright configuration. This yields thicker GL-based magnesicone films, consistent with the experimental observations [24].

DFT studies and experimental work show that aromatic organics can be a better option for the growth of thicker, more flexible and more stable hybrid films. This because aromatic molecules, due to their stiff backbone prefer to orient in a vertical configuration and avoid the double reactions [40,42]. Such hybrid films are known as “metal–organic” films. These also offer the advantage of allowing the facile tuning of the properties of the organic component through ring functionalisation, without impacting on the stability of the resulting films.

Many Ti–organic MLD processes have been developed by using different aromatic molecules as organic precursors. In ref [43], TiCl_4_ was coupled with 4-aminophenol (AP) to deposit Ti–(O–C_6_H_4_–N=) thin films with an essentially ideal growth rate of 10–11 Å/cycle at a temperature range of 120–160 °C. The deposited films were of high quality and stable in air. The high growth rates are attributed to the rigid structure of AP and the fact that Cl ligands of TiCl_4_ are small and do not cause steric hindrance.

In another study, Ti–organic films were deposited using TiCl_4_ and the homo/heterobifunctional aromatic molecules hydroquinone (HQ), 4-aminophenol (AP), *p*-phenylenediamine (PDA) and 4,4′-oxydianiline (ODA). All films were deposited on a Si surface. All four processes yielded amorphous Ti–organic films. A growth rate of 10–11 Å/cycle was achieved for the TiCl_4_–AP process, which is higher when compared to the growth rates 4.3 Å/cycle, 1.2 Å/cycle and 1.4 Å/cycle for TiCl_4_–HQ, TiCl_4_–PDA and TiCl_4_–ODA processes, respectively. The results are attributed the higher reactivity of the OH group with TiCl_4_ in comparison to the NH_2_ group and the higher tendency of the heterobifunctional organic precursor to orientate in an upright configuration and avoid unwanted double reactions on the surface [42].

TiCl_4_ was also coupled with 4,4′-oxydianiline (ODA) to deposit the (–Ti–N–C_6_H_4_–O–C_6_H_4_–N–)*_n_* thin films. Films were deposited on an Si surface in two temperature ranges, 160–230 °C and 250–490 °C. The growth rate increases with increasing temperature, from 0.3 Å/cycle at 160 °C to 1.1 Å/cycle at 490 °C. Films deposited at a higher temperature were also more stable in atmosphere compared to films deposited at low temperatures [44].

Because of the similarity with TiO_2_ ALD films, titanicone films are promising for electronics and solar applications [45] and may be used for biological implants as well. As TiO_2_ films have catalytic and photocatalytic properties [46] porous TiO_2_ frameworks formed by the annealing of titanicone films may serve as catalytic supports [47]. Titanicone films can also be pyrolyzed under Ar to yield conducting TiO_2_/carbon composite films with important electrochemical applications as electrodes for Li ion batteries or pseudocapacitance supercapacitors [31]. These films were also employed as coatings of nano Si electrodes and successfully improved their performance [48].

As described above, different titanicone and Ti–organic MLD processes have been developed and although first principles density functional theory (DFT) simulations have recently been used to explore the reaction mechanism in Ti–organic MLD film growth [42], such a study that explores the surface reactions and the precursor chemistry in titanicone MLD film growth and the role of the precursor chemistries is still lacking.

In this study we investigate the molecular mechanism of formation of titanicone films on anatase TiO_2_, rutile TiO_2_ and Al_2_O_3_ surfaces using TiCl_4_ or Ti(DMA)_4_ as Ti source and EG or GL as organic components. Calculated energetics suggest a higher reactivity of Ti(DMA)_4_ towards the selected surfaces and the organic molecules compared to TiCl_4_. This is due to the stronger Ti–Cl bonds in TiCl_4_ compared to Ti–N bonds in Ti(DMA)_4_. We also found that the choice of surface and inorganic precursors can influence the behavior of organic molecules by allowing or preventing undesirable double reactions. Based on the calculated energetics we propose that a low temperature rutile TiO_2_/TiCl_4_–EG process and rutile TiO_2_/TiCl_4_–GL process can lead to thicker hybrid films.

## Computational Methods

All DFT calculations in this work were performed using the Vienna Ab initio Simulation Package (VASP) version 5.4 [49]. Titanicone films were modelled on an anatase TiO_2_ (101) surface with a coverage of 1 ML OH, rutile TiO_2_ (110) surface with a coverage of 0.75 ML OH, and an Al_2_O_3_ (0001) surface at a coverage 0.50 ML OH [50–51]. These surface models interact with titanium tetrachloride (TiCl_4_) and tetrakis(dimethylamido)titanium (Ti(DMA)_4_) inorganic precursors and then ethylene glycol (EG) and glycerol (GL) organic co-reactants.

The valence electron–core electron interactions are described by projector augmented wave potentials (PAW) [52] and the valance electron configurations are: Ti: 3d^3^4s^1^, Al: 3s^2^3p^1^, O: 2s^2^2p^4^, Cl: 3s²3p⁵, C: 2s^2^2p^2^ and H: 1s^1^. Calculations were performed using the Perdew–Burke–Ernzerhof (PBE) exchange–correlation functional [53]. The employed convergence criterion for the energy was 1 × 10^−4^ eV while that for the forces was −2 × 10^−2^ eV/Å. The geometry was optimized by relaxing the ionic positions using an energy cut-off of 400 eV as well as a Monkhorst−Pack **k**-point sampling grid of (3 × 3 × 1). The lattice parameters are *a* = 20.612, *b* = 15.164, *c* = 24.399 and α = β = γ = 90° for the anatase TiO_2_ surface model, *a* = 11.846, *b* = 13.051, *c* = 33.870 and α = β = γ = 90° for the rutile TiO_2_ surface model and *a* = *b* = 19.228, *c* = 40.627 and α = β = 90°, γ = 120° for the Al_2_O_3_ surface model. The surfaces are 2, 4 and 5 layers thick for anatase (101), rutile (110) and Al_2_O_3_ (0001).

Reaction energetics were calculated using:


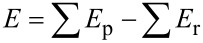


Here *E*_p_ is the energy of products and *E*_r_ is the energy of reactants.

For the example of TiCl_4_ adsorbing on the hydroxylated surfaces the energy was calculated as follows:


[1] $$E_{\text{ads}}=E_{{\text{TiCl}}_{\text{4}}-\text{HO}-\text{surface}}-(E_{{\text{TiCl}}_{\text{4}}}+E_{\text{HO}-\text{surface}}),$$

where 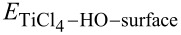 is the total energy of TiCl_4_ adsorbed on hydroxylated surface, 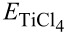 is the total energy of free TiCl_4_ molecule and *E*_HO–surface_ is the total energy of the hydroxylated surface. Energetics of ligand elimination can be calculated from:


[2] $$E_{\text{ads}}=(E_{{\text{TiCl}}_{\text{3}}-\text{O}-\text{surface}}+E_{\text{HCl}})-(E_{{\text{TiCl}}_{\text{4}}-\text{HO}-\text{surface}}),$$

where a HCl molecule is eliminated. A similar equation is used for elimination of another HCl to give TiCl_2_ on the TiO_2_ surface.

For the example of EG interacting on a TiCl_3_-terminated surface the energy was calculated as follows:


[3] $$\begin{array}{r} E_{\text{int}}=(E_{\text{EG}-{\text{TiCl}}_{2}-\text{O}-\text{surface}}+E_{\text{HCl}})\\ -\;(E_{\text{EG}}+E_{{\text{TiCl}}_{\text{3}}-\text{O}-\text{surface}}), \end{array}$$

where 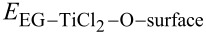 is the total energy of EG bound to TiCl_4_ on the hydroxylated surface, *E*_HCl_ is the total energy of a free HCl molecule released as by-product, *E*_EG_ is the total energy of the EG molecule and 
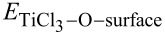
 is the total energy of TiCl_3_-terminated hydroxylated surface. In computing the energies in Equation 1 and Equation 2, we also employ van der Waals interactions using the DFT-D3 parameterisation [54]. Similar energy expressions are used for the Ti(DMA)_4_ precursor.

The underlying data files are available at https://github.com/ArbreshaMuriqi/Titanicone.

## Results and Discussion

### Surface models of TiCl_3_/TiCl_2_-terminated anatase/rutile TiO_2_ and Al_2_O_3_ after the TiCl_4_ pulse

As oxide/metalcone films are of high interest, we explored the feasibility of anatase TiO_2_/titanicone, rutile TiO_2_/titanicone and Al_2_O_3_/titanicone film formation. This is a common approach in modelling MLD chemistry [24,39–41]. We performed fundamental investigation on the interactions between the hydroxylated anatase TiO_2_, hydroxylated rutile TiO_2_ and hydroxylated Al_2_O_3_ surfaces with TiCl_4_ and EG or GL precursors. The hydroxylated surfaces that results from the interactions with water and before the introduction of TiCl_4_ are taken from previous studies [50–51].

In the first calculations we have calculated the interaction energies of the TiCl_4_ molecule on the selected surfaces. These energies are −0.29 eV for the anatase TiO_2_, −0.22 eV for the rutile TiO_2_ and 0.87 eV for the Al_2_O_3_. These energies indicate that TiCl_4_ will adsorb favourably, although with a small energy gain, on the anatase TiO_2_ surface and rutile TiO_2_ surface and will not adsorb on the Al_2_O_3_ surface. A previous study reports that TiO_2_ films grow well using TiCl_4_ and H_2_O on amorphous Al_2_O_3_ [55]. However, in our case, the Al_2_O_3_ surface model we use is crystalline. It is well known that in an amorphous surface the molecular mobility is significantly higher than in any corresponding crystalline form and there is a lower coordination number for atoms in the surface which gives rise to enhanced chemical reactivity of the amorphous surface. These results show that the choice of the surface can have a major impact on the initial deposition steps.

As we found that the adsorption of TiCl_4_ was not favourable on the Al_2_O_3_ surface, we continued with only the anatase TiO_2_ surface models and the rutile TiO_2_ surface models.

**Figure 1:**
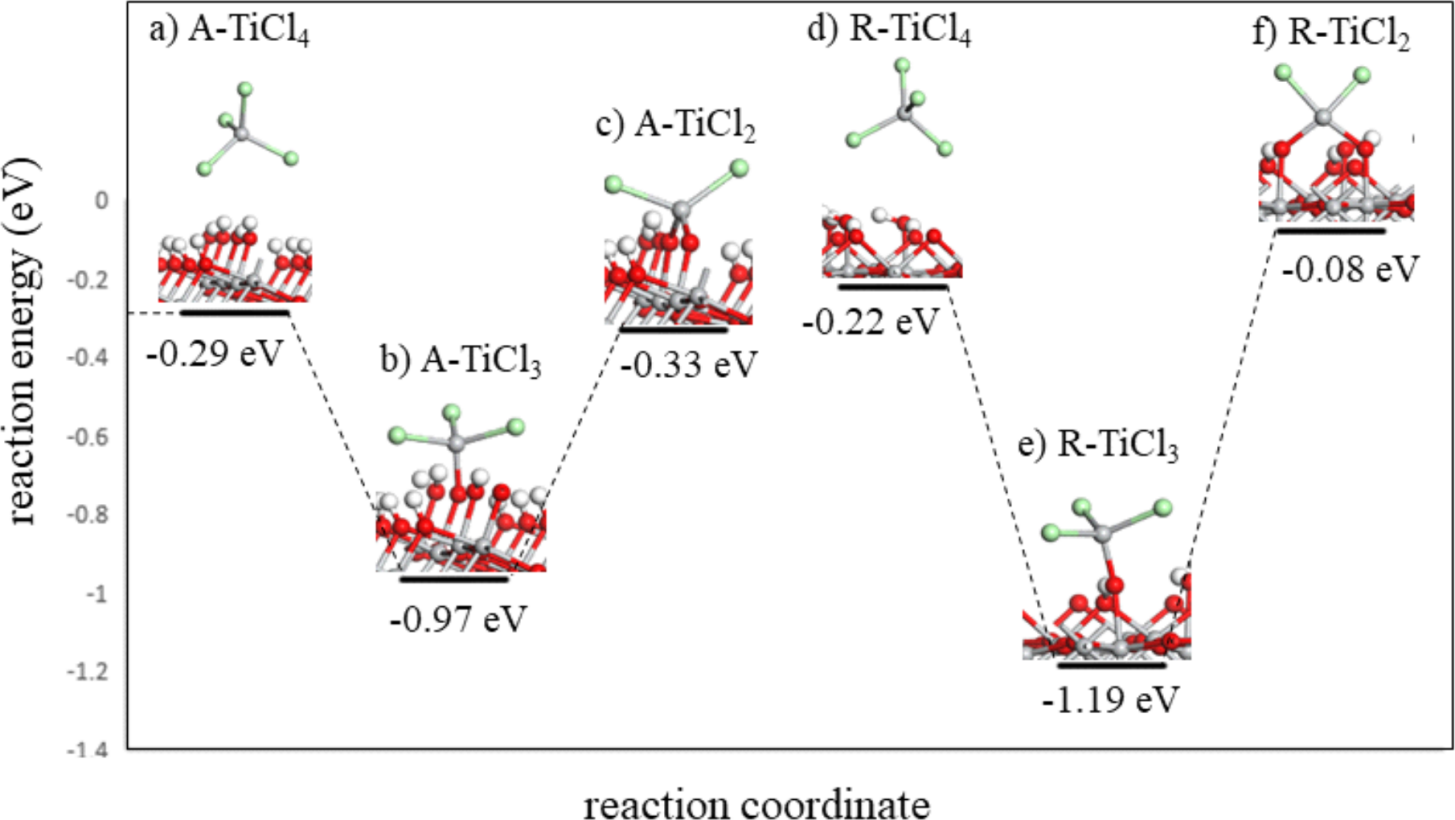
The plotted ligand loss reactions of TiCl_4_ on the anatase/rutile TiO_2_ surfaces. Optimised atomic structure of a) TiCl_4_ interacting with the anatase TiO_2_ surface, b) TiCl_3_-terminated anatase TiO_2_ surface, c) TiCl_2_-terminated anatase TiO_2_ surface, d) TiCl_4_ interacting with the rutile TiO_2_ surface, e) TiCl_3_-terminated rutile TiO_2_ surface and f) TiCl_2_-terminated rutile TiO_2_ surface. Light grey: Ti, red: O, green: Cl, white: H. Figure coding is the same for all figures.

Next, we investigated the first and second ligand loss reactions of TiCl_4_, which involve the proton transfer from the surface OH groups to the Cl ligands of TiCl_4_ to form HCl as by-product, and the formation of new Ti–O bonds between the TiCl_4_ molecule and surface oxygen. During the first ligand loss reaction, TiCl_4_ forms one new Ti–O bond with the surface and one HCl molecule is released. This reaction leaves the surface covered with three Cl ligands, TiO_2_–TiCl_3_. During the second ligand loss reaction TiCl_4_ forms a second new Ti–O bond with the surface and a second HCl molecule is released. After this reaction the surface is left covered with two Cl ligands, TiO_2_–TiCl_2_. The coordination number of Ti in the TiCl_4_ molecule is four and remains unchanged during the first and second ligand loss reactions. Atomic structures of the anatase TiO_2_ surface and rutile TiO_2_ surface after adsorption of one TiCl_4_ molecule and the elimination of the first and second ligand of TiCl_4_ are presented in Figure 1**.** Calculated interaction and ligand loss energies of TiCl_4_ on the anatase TiO_2_ surface and rutile TiO_2_ surface are presented in Table 1**.** Energetics for the ligand loss reactions are calculated relative to the first model of TiCl_4_ interacting with the surface, and present an overall reaction energy. The overall energy for TiCl_4_ to lose Cl ligands and bind on either TiO_2_ surface is negative, although there is a notable energy cost for losing the second Cl ligand from surface bound TiCl_3_. We do not explore further loss of HCl to give adsorbed TiCl since XPS analysis from previous experimental work showed that chlorine impurities are still present in the film [33], indicating that some Ti–Cl bonds have reminded unreacted.

**Table 1:** Computed adsorption and ligand loss energies of TiCl_4_ on anatase/rutile TiO_2_ surfaces. In this and all tables in this paper A: anatase TiO_2_, R: rutile TiO_2_.

Structure	Interaction energy (eV)	Structure	Interaction energy (eV)

A–TiCl_4_	−0.29	R–TiCl_4_	−0.22
A–TiCl_3_	−0.97	R–TiCl_3_	−1.19
A–TiCl_2_	−0.33	R–TiCl_2_	−0.08

The length of the Ti–O bonds formed between TiCl_4_ and both TiO_2_ surfaces during the first and second ligand loss reactions is presented in Table S1 (Supporting Information File 1). From Table S1 we can see that Ti–O bonds formed between anatase TiO_2_ surface and TiCl_4_ are about 0.02–0.03 Å shorter when compared to the Ti–O bonds formed between rutile TiO_2_ surface and TiCl_4_. Generally, shorter Ti–O bonds tend to be stronger, and this can also influence the stability of the system.

In addition to the surface models that result from the adsorption of a single TiCl_4_ molecule, in order to investigate the double reaction phenomenon of EG and GL, we have also built models in which two TiCl_4_ molecules are adsorbed on the anatase TiO_2_ and rutile TiO_2_ surfaces. Calculated energies for the adsorption of the second TiCl_4_ molecule are −0.81 eV on the anatase TiO_2_ surface and −0.03 eV on the rutile TiO_2_ surface. This difference may arise from the higher stability of rutile TiO_2_ compared to anatase TiO_2_. The resulting surfaces after the adsorption of the second TiCl_4_ molecule are terminated with two TiCl_3_ species, anatase–2TiCl_3_ and rutile–2TiCl_3_, and the distance between the Ti atoms of the two TiCl_3_ species is 6.0 Å on the anatase TiO_2_ surface and 6.9 Å on rutile TiO_2_ surface.

### Reactions between organic precursors and TiCl_3_/TiCl_2_-terminated anatase/rutile TiO_2_ surface

**Figure 2:**
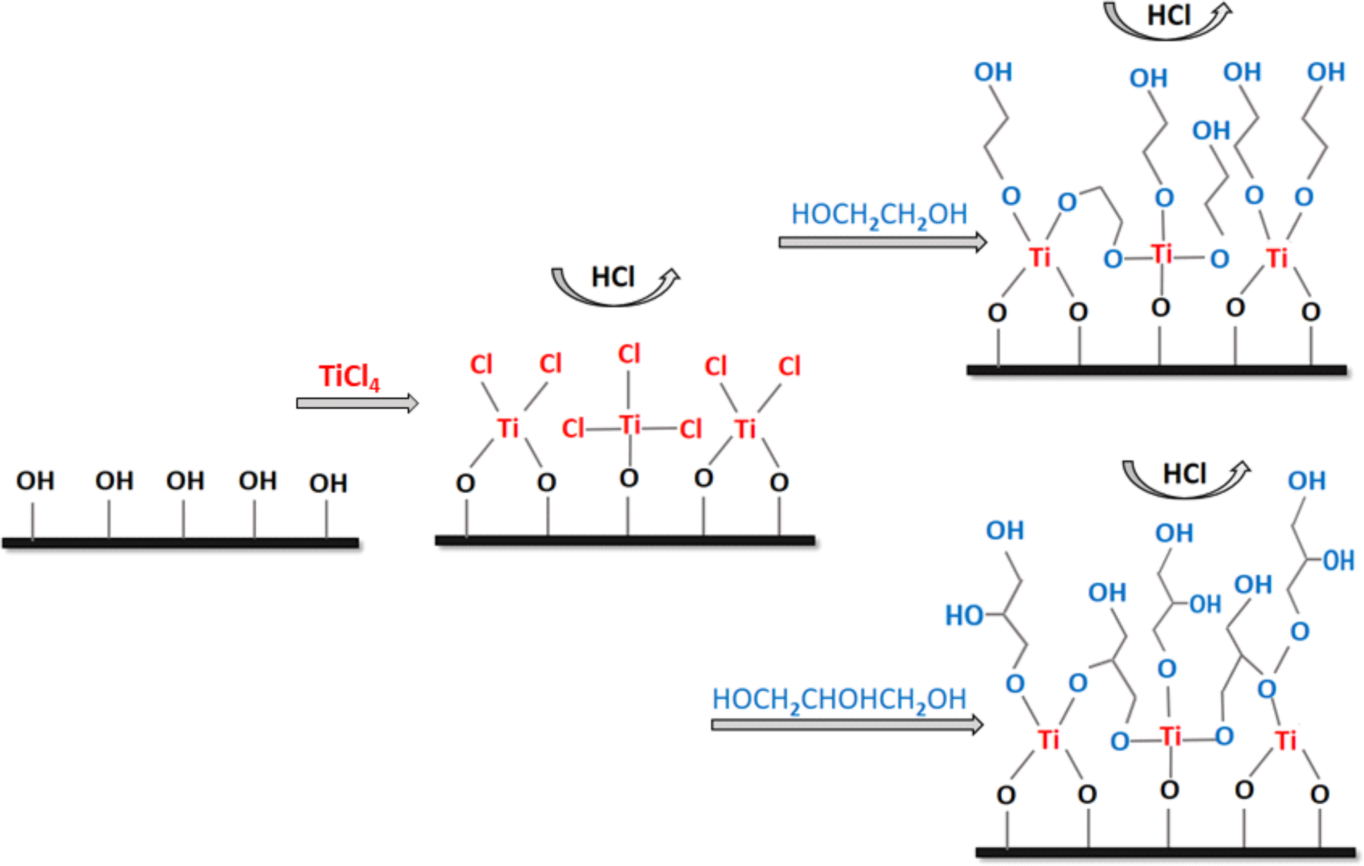
Schematic representation of titanicone MLD using titanium tetrachloride (TiCl_4_) as inorganic precursor and ethylene glycol (EG) or glycerol (GL) as organic precursors.

**Figure 3:**
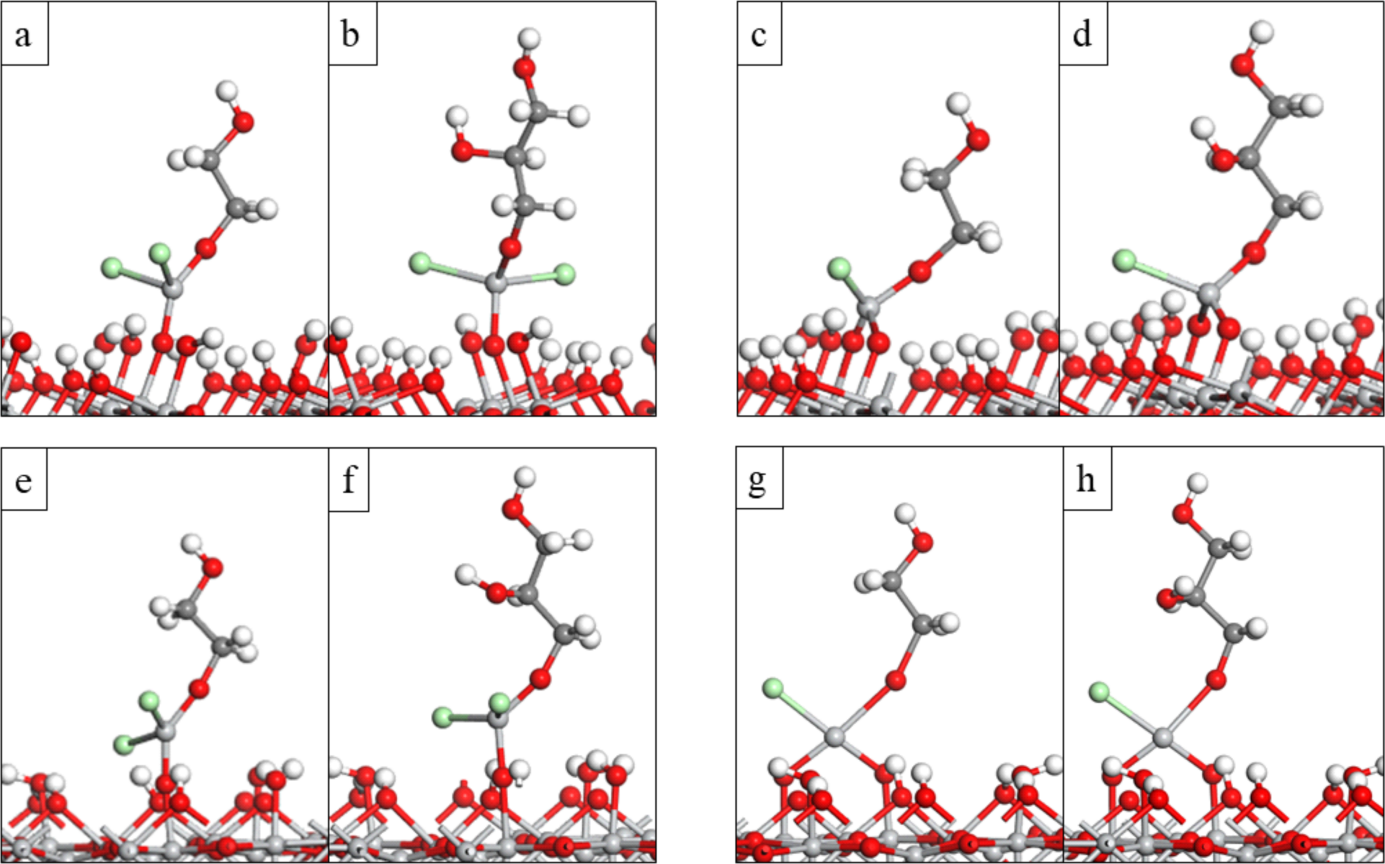
Optimised atomic structure of a) anatase TiCl_3_–EG, b) anatase TiCl_3_–GL, c) anatase TiCl_2_–EG, d) anatase TiCl_2_–GL, e) rutile TiCl_3_–EG, f) rutile TiCl_3_–GL, g) rutile TiCl_2_–EG and h) rutile TiCl_2_–GL.

With these post-TiCl_4_ pulse models of anatase TiO_2_ and rutile TiO_2_ available, the interactions between the TiO_2_–TiCl_3_ and TiO_2_–TiCl_2_ species and the organic precursors are investigated by analysing the formation of MLD products with EG and GL. Reactions with EG and GL involve the transfer of one proton from a terminal OH group of the organic molecule to a Cl ligand of TiCl_3_/TiCl_2_ to release a HCl molecule as by-product and form one new Ti–O bond between the TiCl_3_/TiCl_2_ species and the organic molecules. A schematic illustration of titanicone MLD films based on the reactions between the hydroxylated surface and TiCl_4_ and the reactions between the TiCl_3_/TiCl_2_ surface species with EG and GL is presented in Figure 2. The resulting atomic structures of the MLD reaction products with EG and GL are shown in Figure 3.

The overall energy change for the reactions between the TiCl_3_/TiCl_2_-terminated anatase TiO_2_ and rutile TiO_2_ surfaces with EG and GL is presented in Table 2 and these results show that the calculated energies for the reactions between the TiCl_3_-terminated surfaces with EG and GL, associated with the release of one HCl molecule, are exothermic and therefore favourable. There are also clear differences when EG and GL bind with TiCl_3_ and TiCl_2_-terminated TiO_2_ surfaces. The calculated energies for the reactions between the TiCl_2_-terminated surfaces with EG and GL, associated with the release of one HCl molecule, are endothermic, meaning that these reactions are not favourable. We found a similar difference for the reaction of TiCl_3_/TiCl_2_ with aromatic precursors [42].

**Table 2:** Computed interaction energies of EG and GL on the TiCl_3_/TiCl_2_-terminated anatase/rutile TiO_2_ surface.

Structure	Interaction energy (eV)	Structure	Interaction energy (eV)

A–TiCl_3_–EG	−0.79	R–TiCl_3_–EG	−0.96
A–TiCl_3_–GL	−0.66	R–TiCl_3_–GL	−1.77
A–TiCl_2_–EG	0.08	R–TiCl_2_–EG	0.49
A–TiCl_2_–GL	0.15	R–TiCl_2_–GL	0.81

We also consider the Ti–O distances formed between the EG and GL with TiCl_3_/TiCl_2_ species as well as changes in the Ti-surface distances between TiCl_3_ and TiCl_2_ and surface oxygens after the introduction of EG and GL, Table S2 (Supporting Information File 1). We notice that Ti–O bonds formed between EG and GL and the TiCl_3_ terminated anatase TiO_2_ and rutile TiO_2_ surfaces are 0.02 Å shorter compared to Ti–O bonds formed between EG and GL with the TiCl_2_ terminated anatase TiO_2_ and rutile TiO_2_ surfaces. Ti–O distances between TiCl_4_ and the surface oxygens also change after the introduction of EG and GL. After the introduction of EG and GL, Ti–O bonds to the surface are lengthen for 0.02 Å to 0.03 Å in the TiCl_3_ terminated anatase TiO_2_ and rutile TiO_2_ surfaces and 0.02 Å to 0.07 Å in the TiCl_2_ terminated anatase TiO_2_ and rutile TiO_2_ surfaces. As DFT results suggest that the formation of Ti–O bonds between EG and GL and the TiCl_2_ species on the anatase TiO_2_ and rutile TiO_2_ surfaces is energetically unfavourable, in the next calculations we exclude the TiCl_2_ terminated surfaces.

### Comparison of upright and flat-lying reactions of ethylene glycol (EG) and glycerol (GL) on the TiCl_3_-terminated anatase/rutile TiO_2_ surface

Double reactions of EG and GL are investigated by examining the interactions between the TiO_2_ surface terminated with 2TiCl_3_ and 2TiCl_3_ with EG and GL in the upright configuration and in the flat configuration. In the upright configuration, EG and GL bind to Ti sites through one terminal OH group and one HCl molecule is released. In the flat configuration, EG and GL bind through two terminal OH groups with two neighbouring Ti sites and two HCl molecules are released.

**Figure 4:**
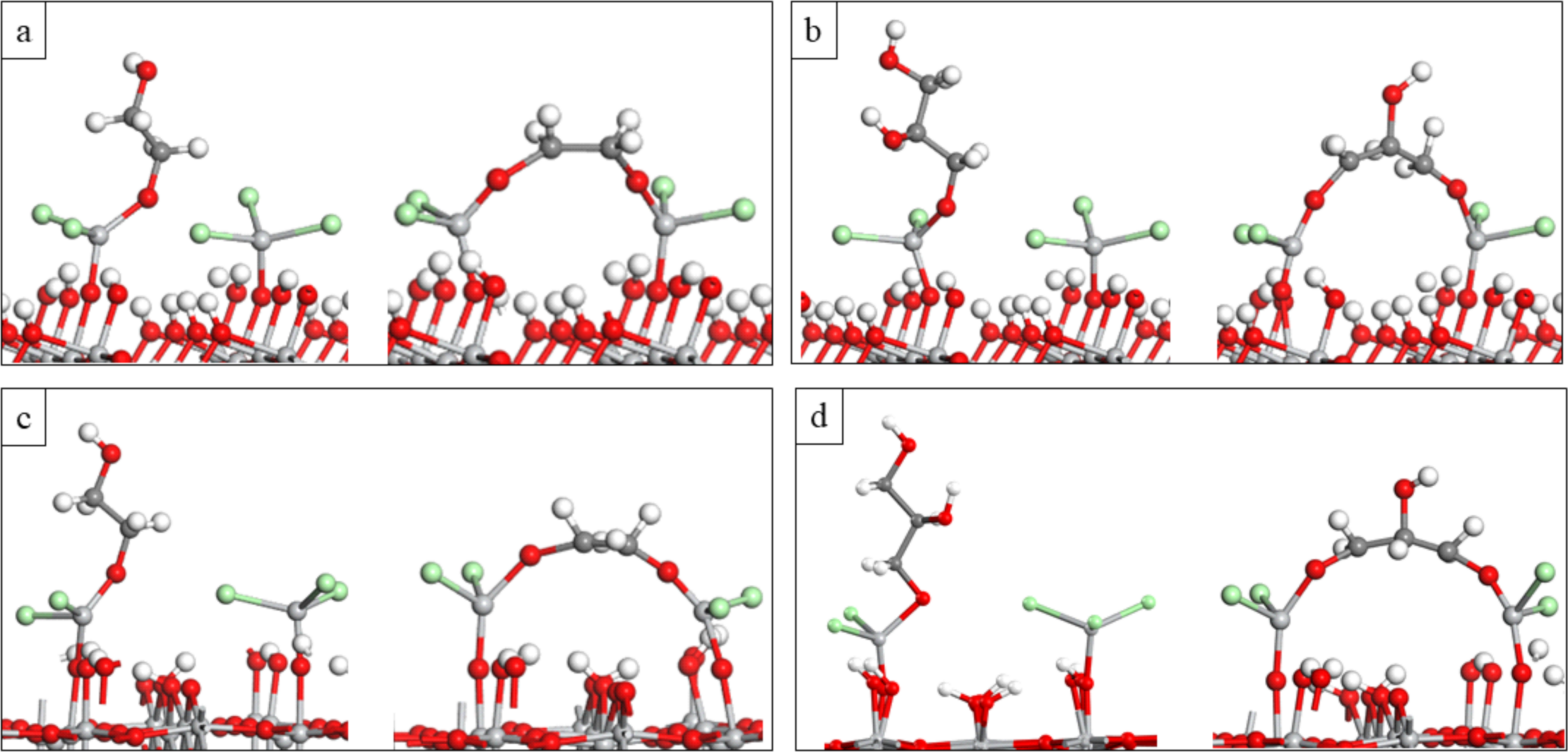
Optimised atomic structure of a) upright and flat EG on the anatase 2TiCl_3_, b) upright and flat GL on the anatase 2TiCl_3_, c) upright and flat EG on the rutile 2TiCl_3_, d) upright and flat GL on the rutile 2TiCl_3_.

Optimised atomic structures of the MLD reaction products of TiCl_3_-terminated anatase TiO_2_ and rutile TiO_2_ surfaces with upright and flat lying EG and GL are shown in Figure 4. The computed energies when EG and GL bind with one Ti site in the upright configuration and with two Ti sites in the flat lying configuration are shown in Table 3. The calculated energy for the upright configuration of EG and GL is calculated with reference to the energy of the adsorbed Ti precursor on the relevant surface, while the calculated energy for the flat configuration of EG and GL is with reference to the energy of the corresponding upright structure. This allows us to assess if the double reactions of EG and GL are thermodynamically favourable when reacting with TiCl_3_ on anatase TiO_2_ and rutile TiO_2_ surfaces.

**Table 3:** Computed interaction energy of EG and GL in the upright configuration with TiCl_3_-terminated anatase/rutile TiO_2_ surface. The energy change between the flat (double reaction) and upright configuration is also presented.

Structure	Interaction energy (eV)	Structure	Interaction energy (eV)

A–2TiCl_3_	−0.81	R–2TiCl_3_	−0.03
A–2TiCl_3_–EG–up	−0.72	R–2TiCl_3_–EG–up	−0.46
A–2TiCl_3_–EG–flat	−0.55	R–2TiCl_3_–EG–flat	0.34
A–2TiCl_3_–GL–up	−0.63	R–2TiCl_3_–GL–up	−0.36
A–2TiCl_3_–GL–flat	−0.33	R–2TiCl_3_–GL–flat	0.33

From Table 3 we see that the EG and GL molecules interact favourably in an upright configuration at the 2TiCl_3_-terminated anatase TiO_2_ surface. However, there is a further gain in energy of −0.55 eV for EG and −0.33 eV for GL when the molecules change their configuration from upright to flat lying, and a second HCl molecule is released. These energies show that EG and GL could also undergo double reactions on the anatase TiO_2_ surface, although for GL this difference in energy is smaller than for EG.

Similar to Al_2_O_3_, this phenomenon for EG will reduce the number of OH sites on the surface and this should make the growth of TiCl_4_–EG films less favourable. However, despite the reduction of OH groups on the surface, terminal oxygen sites of the flat EG can also serve as active sites and they can bind with other TiCl_4_ molecules in the following cycle, similar to TMA–EG alucones [39]. The GL molecule is a triol with three OH active groups, so even in the case of double reactions for GL the third OH group is available for further reactions.

The EG and GL molecules interact also favourably in an upright configuration with the 2TiCl_3_-terminated rutile TiO_2_ surface, while for their flat lying configuration the overall energy change is slightly endothermic. This shows that in a rutile surface EG and GL could prefer to orient in an upright configuration. For the 2TiCl_3_–EG model, the distance between Ti and O in the Ti–O–CH_2_CH_2_–O fragment that is formed during one MLD cycle (EG in the upright configuration) is ≈4.97 Å. This distance is similar with the achieved growth rate of ≈4.5 Å/cycle to 6 Å/cycle for TiCl_4_–EG films in a temperature range of 100–120 °C on a SiO_2_ membrane [27] and ≈4.6 Å/cycle at 90 °C in Si(100) wafers [30]. We note the relatively low temperature of the titanicone deposition in refs. [27,30] which may prevent the double reaction with EG.

For the TiCl_3_–GL model, the distance between Ti and O in the Ti–O–CH_2_CH_2_OHCH_2_–O fragment (GL in the upright configuration) is ≈6.26 Å. However, the achieved growth rate of TiCl_4_–GL films in experimental work is much smaller, 2.8 Å/cycle at 130 °C to 2.1 Å/cycle at 210 °C when deposited in Si wafers [30] and 2.2 Å/cycle at 150 °C deposited again in Si wafers in another study [31]. The distance between the surface and the available OH group of the flat GL is 4.2 Å and this distance is close to the measured growth per cycle of TiCl_3_–GL films in experimental work. Although the lower growth per cycle of TiCl_3_–GL films achieved in these experimental studies are most consistent with a flat lying orientation of GL when deposited on Si surfaces, DFT shows that the desired upright orientation of GL could be energetically favourable on a rutile TiO_2_ surface and this provides motivation to develop a lower temperature rutile TiO_2_/TiCl_4_–GL process where higher growth per cycle could be achieved. We note that the experimental data are not immediately comparable since different deposition temperatures are used, with significantly higher temperatures for the GL process compared to the EG process which may favour the flat-lying configuration.

In summary, DFT calculations show that while in a anatase TiO_2_ surface the organic molecules EG and GL can orient in both configurations, upright and lying flat, in the rutile TiO_2_ surface these molecules could prefer more the upright configuration, although the flat-lying configuration may also be stabilised. This indicates that the surface can also have an important role on the orientation of the organic species.

The Ti–O distances to the EG and GL in the upright and flat lying configurations are presented in Table S3 (Supporting Information File 1). The computed distances show that the Ti–O bond to the molecules does not change significantly when EG and GL change their configuration from upright to lying flat on the anatase TiO_2_ surface (0.01 Å longer). On the other hand, on rutile, the Ti–O bonds to the organic molecules in the flat lying configuration are 0.03 Å to 0.06 Å longer compared to Ti–O bonds of these molecules in the upright configuration. The increment of these bonds when EG and GL change their configuration from upright to lying flat on the rutile TiO_2_ surface also indicates the lower stability of these molecules in the flat configuration on the TiCl_3_ terminated rutile TiO_2_ surface.

For the upright EG and GL on the anatase TiO_2_ surface, the Ti–O distance between Ti of TiCl_4_ and the oxygen atom of the anatase TiO_2_ surface is 1.77 Å. This distance decreases to 1.75 Å when EG and GL change their configuration to lying flat. For EG in the upright and flat laying configuration on the rutile TiO_2_ surface the Ti–O distance between Ti of TiCl_4_ and the oxygen atom is 1.77 Å. For GL in the upright and flat lying configuration on the rutile TiO_2_ surface this distance is 1.76 Å.

### Surface models of Ti(DMA)_3_, Ti(DMA)_2_ and TiDMA-terminated anatase/rutile TiO_2_ and Al_2_O_3_ after the Ti(DMA)_4_ pulse

Ti(DMA)_4_ is another Ti precursor that has been used for the deposition of TiO_2_ [35] and TiN [36] ALD films and titanicone MLD films [33]. Ti(DMA)_4_ is a metalorganic precursor containing Ti–N bonds to the organic ligands, dimethylamino (DMA, N(CH_3_)_2_), and with a much larger molecular size when compared to TiCl_4_. It offers some advantages as a precursor, including the noncorrosive nature of by-products of ligand elimination, higher reactivity due to the weak Me–N bonds and good thermal stability [38]. We performed DFT calculations to study the feasibility of the growth of titanicone films using Ti(DMA)_4_ and EG or GL as MLD precursors and to investigate the molecular mechanism behind the possible growth. The hydroxylated anatase TiO_2_ surface, hydroxylated rutile TiO_2_ surface and hydroxylated Al_2_O_3_ surface were selected as surface models.

In the first calculations, we calculate the reactivity of the Ti(DMA)_4_ precursor towards the selected surfaces. The calculated interaction energies on the anatase TiO_2_, rutile TiO_2_ and the Al_2_O_3_ surfaces are −0.88 eV, −0.74 eV and −1.47 eV, respectively. Thus, Ti(DMA)_4_ adsorbs favourably on the three oxide surfaces.

Next, the thermodynamics of the first, second and third ligand loss reactions of the Ti(DMA)_4_ precursor are examined. These reactions take place with a proton transfer from surface OH groups to the DMA ligands of Ti(DMA)_4_ to form protonated molecules, H–DMA, released as by-products and the formation of new Ti–O bonds between Ti(DMA)_4_ and oxygen atoms on the surface. After the third ligand loss reaction, Ti of Ti(DMA)_4_ is bonded to three surface oxygens and to one DMA ligand that can exchange with one organic molecule during the organic precursor pulse. The coordination number of Ti of the Ti(DMA)_4_ molecule is four and remains unchanged during the ligand loss reactions.

**Figure 5:**
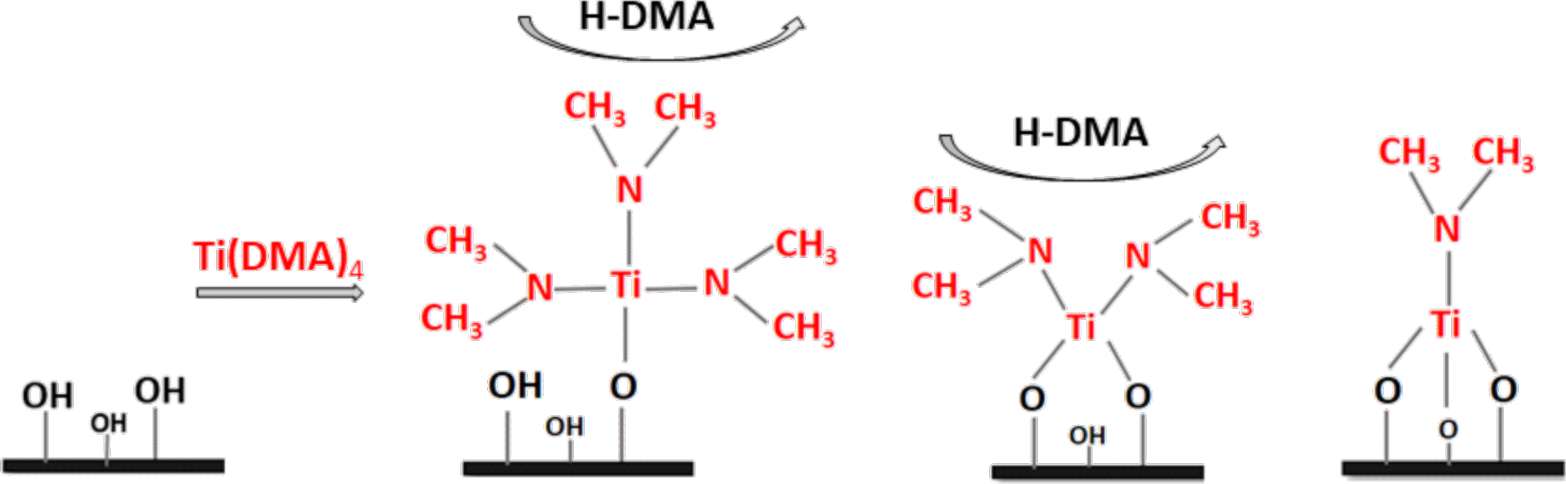
Schematic representation of ligand loss reactions of tetrakis(dimethylamido)titanium (Ti(DMA)_4_) inorganic precursor on a hydroxylated surface.

**Figure 6:**
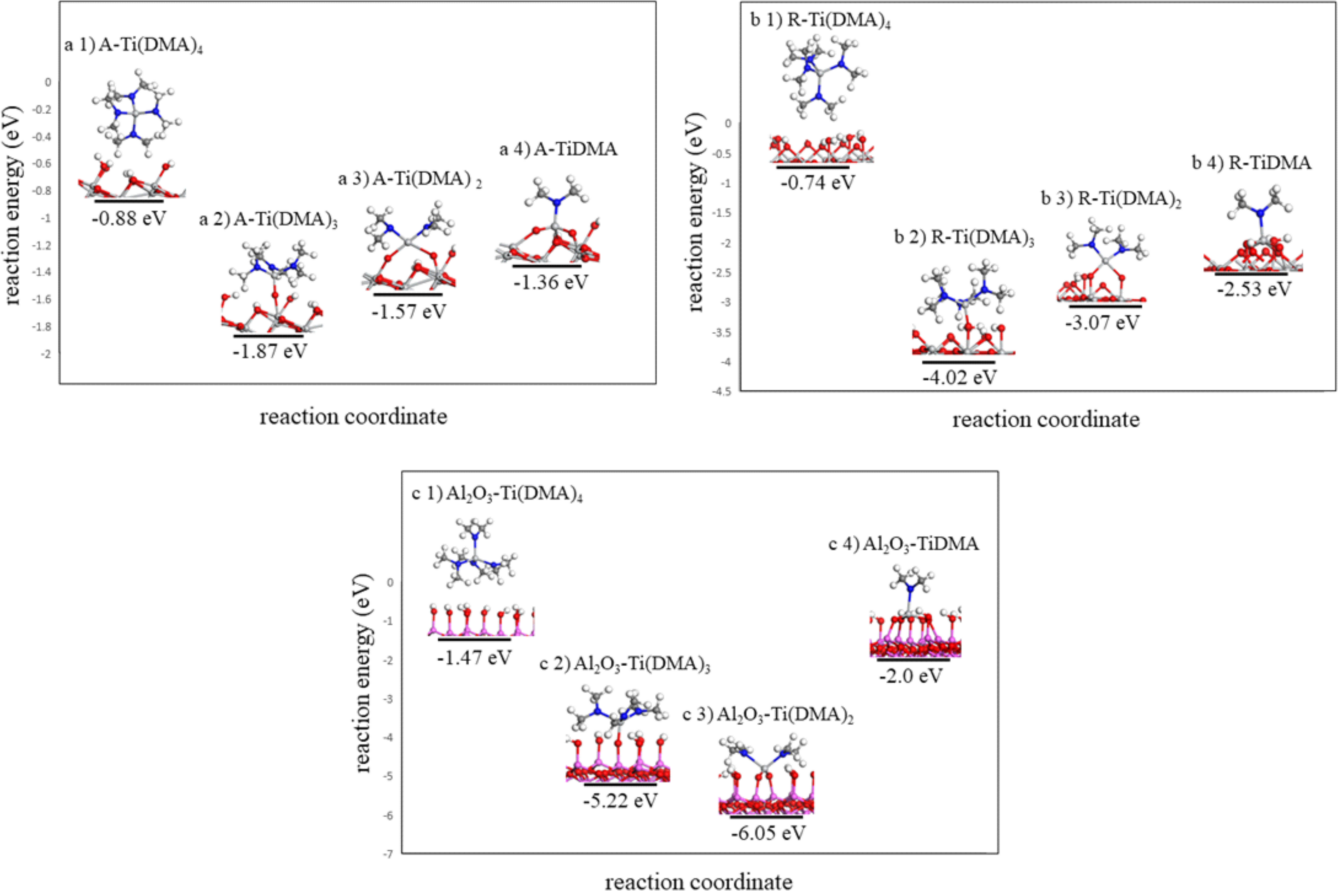
The plotted ligand loss reactions of Ti(DMA)_4_ on the anatase/rutile TiO_2_ surface and Al_2_O_3_ surface. Optimised atomic structure of a1) Ti(DMA)_4_ interacting with the anatase TiO_2_ surface, a2) Ti(DMA)_3_-terminated anatase TiO_2_ surface, a3) Ti(DMA)_2_-terminated anatase TiO_2_ surface, a4) TiDMA-terminated anatase TiO_2_ surface, b1) Ti(DMA)_4_ interacting with the rutile TiO_2_ surface, b2) Ti(DMA)_3_-terminated rutile TiO_2_ surface, b3) Ti(DMA)_2_-terminated rutile TiO_2_ surface, b4) TiDMA-terminated rutile TiO_2_ surface, c1) Ti(DMA)_4_-interacting with the Al_2_O_3_ surface, c2) Ti(DMA)_3_-terminated Al_2_O_3_ surface, c3) Ti(DMA)_2_-terminated Al_2_O_3_ surface and c4) TiDMA-terminated Al_2_O_3_ surface. Dark grey: C, blue: N. Figure coding is the same for all figures.

The ligand loss reaction mechanism of Ti(DMA)_4_ is presented in Figure 5. Optimized atomic structures of the anatase TiO_2_, rutile TiO_2_ and the Al_2_O_3_ surfaces interacting with Ti(DMA)_4_ and after the elimination of the first, second and third ligand of Ti(DMA)_4_ are presented in Figure 6. The computed interaction and ligand loss energies of Ti(DMA)_4_ are presented in Table 4. The energies for the ligand loss reactions are calculated relative to the first model of Ti(DMA)_4_ interacting with the surface, and present an overall reaction energy. Ligand loss energies show that the overall energy for Ti(DMA)_4_ to lose DMA ligands and bind on either TiO_2_ surface or Al_2_O_3_ surface is favourable.

**Table 4:** Computed adsorption and ligand loss energies of Ti(DMA)_4_ on the anatase/rutile TiO_2_ surface and Al_2_O_3_ surface.

Structure	Interaction energy (eV)	Structure	Interaction energy (eV)	Structure	Interaction energy (eV)

A–Ti(DMA)_4_	−0.88	R–Ti(DMA)_4_	−0.74	Al_2_O_3_–Ti(DMA)_4_	−1.47
A–Ti(DMA)_3_	−1.87	R–Ti(DMA)_3_	−4.02	Al_2_O_3_–Ti(DMA)_3_	−5.22
A–Ti(DMA)_2_	−1.57	R–Ti(DMA)_2_	−3.07	Al_2_O_3_–Ti(DMA)_2_	−6.05
A–TiDMA	−1.36	R–TiDMA	−2.53	Al_2_O_3_–TiDMA	−2

When we compare these energies to those calculated for TiCl_4_ interacting and binding on the same selected surfaces, we see that Ti(DMA)_4_ interacts and binds more favourably with the surfaces as the calculated reaction energies are much larger. This is consistent with bond dissociation energy values for the breakage of the Ti–Cl and Ti–N bonds (494 kJ/mol and 464 kJ/mol, respectively) which show that the Ti–Cl bond is stronger and thus more difficult to break.

The overall reaction energy which includes the interaction of Ti(DMA)_4_ with the surface and the three ligand loss reactions is the largest on the rutile TiO_2_ surface, −2.53 eV, which is −0.53 eV larger when compared to the Al_2_O_3_ surface and −1.73 eV larger when compared to the anatase TiO_2_ surface.

The new Ti–O bonds of Ti(DMA)_4_ to the Al_2_O_3_ surface are 0.03–0.08 Å shorter, when compared to those formed between Ti(DMA)_4_ and the anatase TiO_2_ surface or rutile TiO_2_ surface, Table S4 (Supporting Information File 1).

In order to investigate double reactions of EG and GL we built also the surface models after the adsorption of two Ti(DMA)_4_ molecules. The reactions when a second Ti(DMA)_4_ molecule is adsorbed are associated with the release of three H-DMA molecules as by-products and the surfaces are left covered with two TiDMA species, anatase–2TiDMA, rutile–2TiDMA and Al_2_O_3_–2TiDMA. Calculated energetics for the adsorption of the second Ti(DMA)_4_ precursors are −1.05 eV for the anatase TiO_2_ surface, −1.29 eV for the rutile TiO_2_ surface, −2.06 eV for the Al_2_O_3_ surface. The distance between Ti atoms of the two TiDMA species is 7.2 Å on the anatase TiO_2_ surface and rutile TiO_2_ surface and 7.1 Å on the Al_2_O_3_ surface.

### Reactions between organic precursors and TiDMA-terminated anatase/rutile TiO_2_ and Al_2_O_3_ surfaces

**Figure 7:**
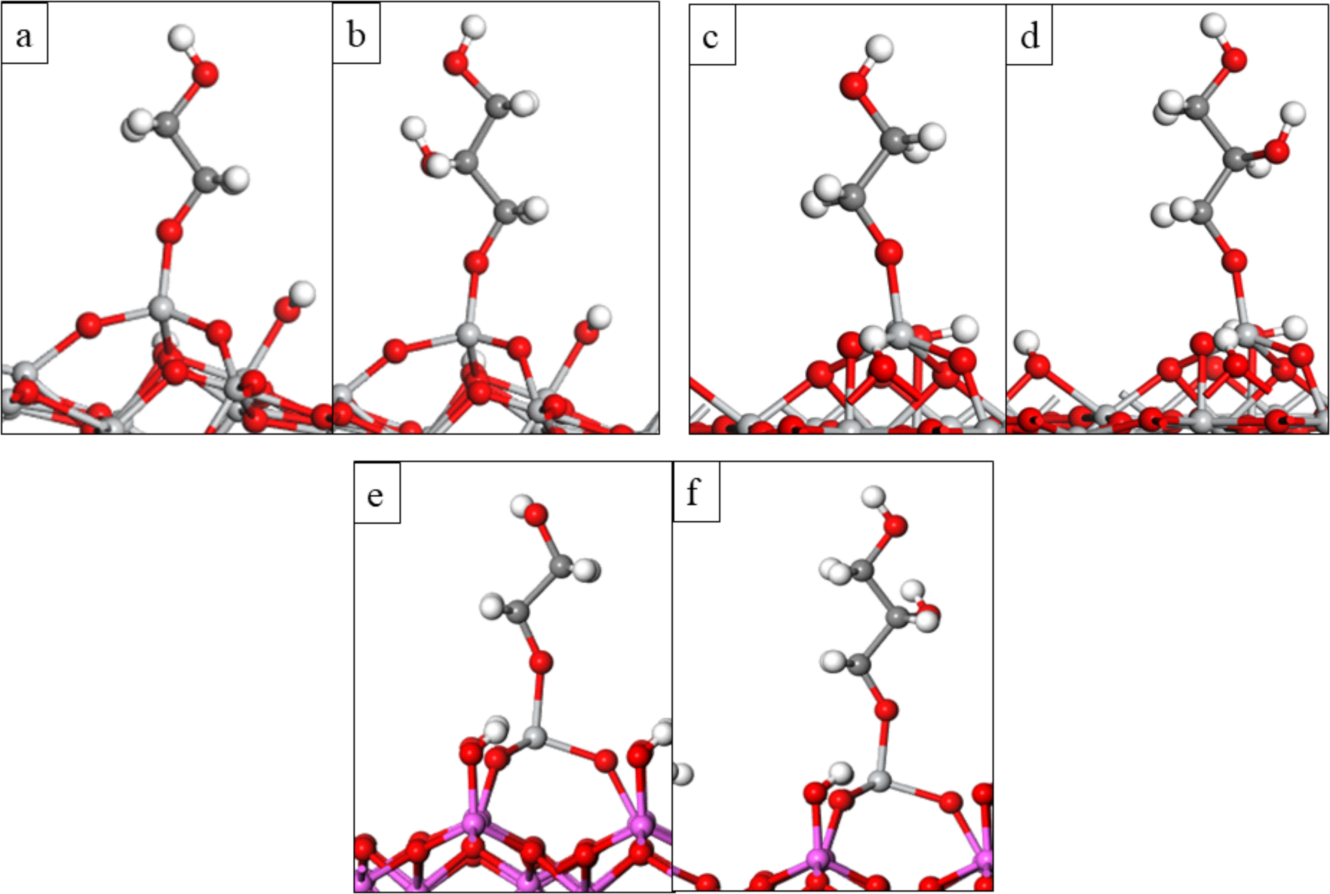
Optimised atomic structure of a) anatase TiDMA–EG, b) anatase TiDMA–GL, c) rutile TiDMA–EG, d) rutile TiDMA–EG, e) Al_2_O_3_ TiDMA–EG and f) Al_2_O_3_ TiDMA–GL.

Next, we analysed MLD reactions using EG and GL as organic reactants where the organic molecules are modelled in both upright and flat lying configurations. Similar to the reaction between TiCl_4_ and EG or GL, the reaction between Ti(DMA)_4_ and EG or GL requires the transfer of one proton from the terminal OH group of the organic molecule to one DMA ligand of Ti(DMA)_4_ to release a H–DMA molecule as a by-product coupled with the formation of a new Ti–O bond between Ti and the organic molecule. Figure 7 shows the optimized atomic structures after the introduction of one EG and GL molecule and the loss of one H–DMA by-product.

The overall energy change for the reactions between the TiDMA**-**terminated anatase TiO_2_, rutile TiO_2_ and Al_2_O_3_ surfaces with EG and GL is presented in Table 5. Calculated energies show that the Ti–O bond formation with EG and GL, with release of H–DMA, is favourable on all surfaces. The overall interaction energy is largest on anatase TiO_2_ followed by rutile TiO_2_.

**Table 5:** Computed interaction energies of EG and GL on the TiDMA-terminated anatase/rutile TiO_2_ and Al_2_O_3_ surfaces.

Structure	Interaction energy (eV)	Structure	Interaction energy (eV)	Structure	Interaction energy (eV)

A–TiDMA–EG	−3.42	R–TiDMA–EG	−2.98	Al_2_O_3_–TiDMA–EG	−1.75
A–TiDMA–GL	−3.31	R–TiDMA–GL	−2.89	Al_2_O_3_–TiDMA–GL	−1.60

Ti–O distances between Ti and the organic molecules are shown in Table S5 (Supporting Information File 1). Ti–O bonds formed between EG and GL with the TiDMA-terminated anatase TiO_2_ and rutile TiO_2_ surfaces are 0.06 Å to 0.08 Å shorter when compared to those formed between the EG and GL and the TiDMA-terminated Al_2_O_3_ surface, which is consistent with the more favourable interaction energies.

We also examined the change in the Ti–O distances to the surface oxygens after the introduction of the EG and GL, and these are presented in Table S6 (Supporting Information File 1). The computed Ti–O distances show that after the introduction of the EG and GL Ti–O bonds to the surface oxygens undergo negligible changes, indicating that the EG and GL will not affect the stability of the systems.

### Comparison of upright and flat-lying reactions of ethylene glycol (EG) and glycerol (GL) on the 2TiDMA-terminated anatase/rutile TiO_2_ and Al_2_O_3_ surfaces

**Figure 8:**
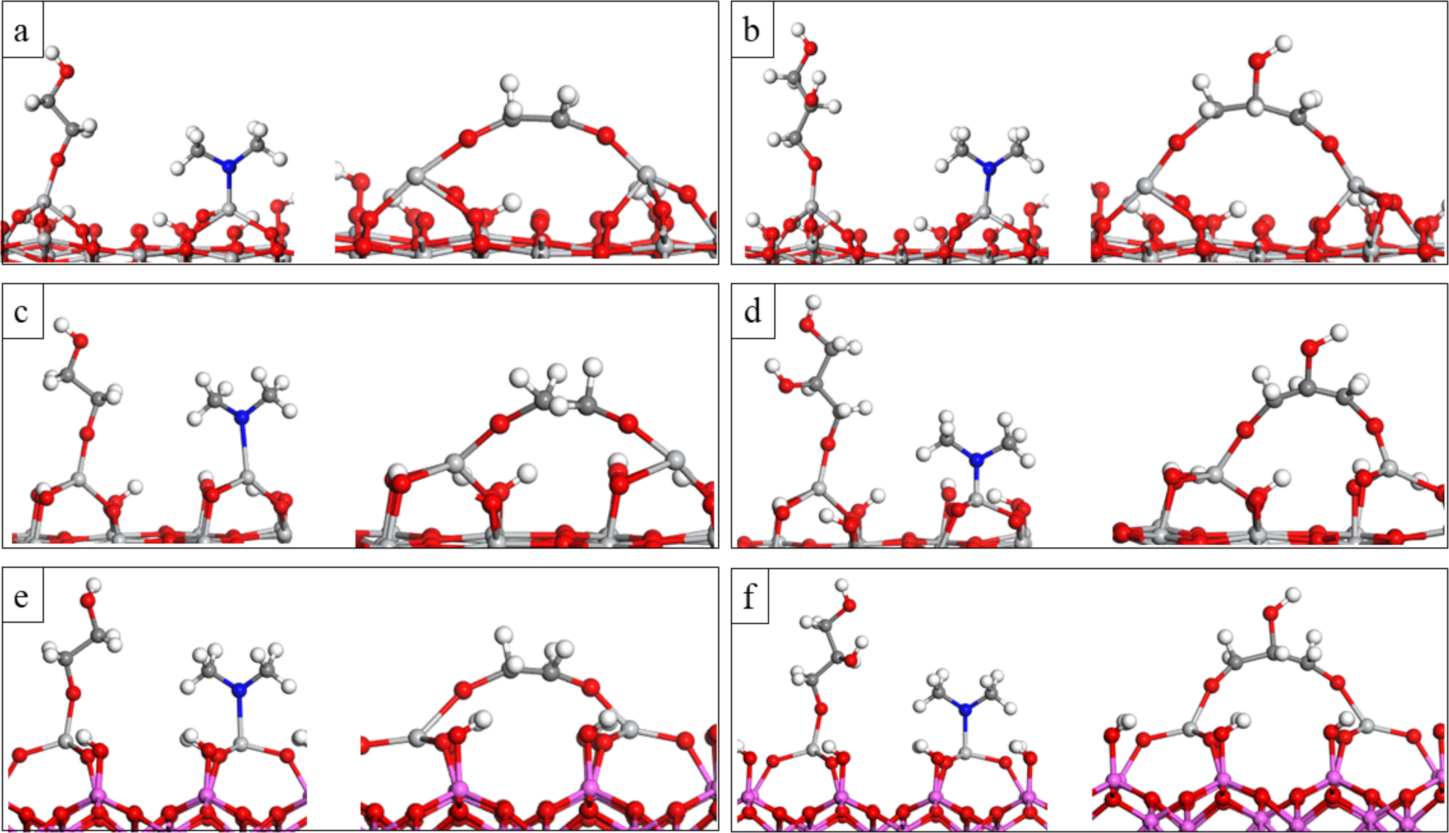
Optimised atomic structure of a) upright and flat EG on the anatase–2TiDMA, b) upright and flat GL on the anatase–2TiDMA, c) upright and flat EG on the rutile–2TiDMA, d) upright and flat GL on the rutile–2TiDMA, e) upright and flat EG on the Al_2_O_3_–2TiDMA and f) upright and flat GL on the Al_2_O_3_–2TiDMA.

We investigated also the double reactions of EG and GL on the anatase-2TiDMA surface, rutile–2TiDMA surface and Al_2_O_3_–2DMA, where EG and GL are modelled in the upright and flat lying configurations. In the upright configuration organic molecules bind to Ti sites through one terminal OH group and one H–DMA molecule is released while in the flat configuration organic molecules will bind through two terminal OH groups with two neighbouring Ti sites and two H–DMA molecules are released. Figure 8 shows the optimised atomic structures of the MLD reaction products of 2TiDMA-terminated anatase TiO_2_, rutile TiO_2_ and Al_2_O_3_ surfaces with the upright and flat lying EG and GL. Calculated energetics for the reactions of EG and GL in the upright and flat laying configuration with the 2TiDMA-terminated surfaces are shown in Table 6.

**Table 6:** Computed interaction energies of EG and GL in the upright configuration with the 2TiDMA-terminated anatase/rutile TiO_2_ and Al_2_O_3_ surfaces. The energy change between the flat (double reaction) and upright configuration is also presented.

Structure	Interaction energy (eV)	Structure	Interaction energy (eV)	Structure	Interaction energy (eV)

A– 2TiDMA	−1.05	R–2TiDMA	−1.29	Al_2_O_3_–2TiDMA	−2.06
A–2TiDMA–EG–up	−1.44	R–2TiDMA–EG–up	−1.65	Al_2_O_3_–2TiDMA–EG–up	−1.73
A–2TiDMA–EG–flat	−0.32	R–2TiDMA–EG–flat	−0.20	Al_2_O_3_–2TiDMA–EG–flat	−1.6
A–2TiDMA–GL–up	−1.40	R–2TiDMA–GL–up	−1.61	Al_2_O_3_–2TiDMA–GL–up	−1.81
A–2TiDMA–GL–flat	−0.68	R–2TiDMA–GL–flat	−0.90	Al_2_O_3_–2TiDMA–GL–flat	−1.3

Calculated energetics for the reactions between upright EG and GL with the 2TiDMA-terminated anatase TiO_2_, rutile TiO_2_ and Al_2_O_3_ surfaces are negative, meaning that the reactions are exothermic and therefore favourable. The energies for the reactions between the upright EG and GL and the 2TiDMA-terminated anatase TiO_2_, rutile TiO_2_ and Al_2_O_3_ surfaces are calculated relative to the energy for the adsorption of two Ti(DMA)_4_ molecules on the relevant surface. The energies for the flat configuration are calculated relative to the upright structures of EG and GL. A negative energy gain was calculated for all reactions when EG and GL change their configuration from upright to flat lying and one H–DMA molecule is released, regardless of the surface.

Calculated energetics show that while the EG and GL can bind to the Ti sites via formation of Ti–O bonds and loss of H–DMA, it is most favourable for the organic precursors to lie flat and create the double reactions through the two terminal OH groups. This phenomenon removes all active sites for EG and the growth will be less favourable while GL has an extra OH group compared to EG which reacts with Ti(DMA)_4_ in the next cycle and the growth proceeds.

Calculations energetics suggest that organic molecules EG and GL combined with Ti(DMA)_4_ in a anatase TiO_2_, rutile TiO_2_ and Al_2_O_3_ surface behave similarly as when combined with Ti(DMA)_4_ on a Si substrate [33]. Experimental data in ref [33] show that for Ti(DMA)_4_–EG films deposited on a Si substrate in a temperature range of 80–150 °C the growth starts but it stops after 5–10 cycles, and this most probably due to the favourable double reactions of EG molecules with the Ti species. For Ti(DMA)_4_–GL films the growth proceeds even when the molecule reacts twice with the surface due to the extra OH group which reacts with Ti(DMA)_4_ in the next cycle. However, although the growth of Ti(DMA)_4_–GL films proceeds, these double reactions lead to small GPCs, 0.9 Å/cycle to 0.2 Å/cycle when deposited in a Si substrate in a temperature range of 80–160 °C. The stability and GPC of the Ti(DMA)_4_–GL-based film may also depend on the strength of interaction with GL, which is less favourable than with EG.

The Ti–O distances to the EG and GL molecules in the upright and flat lying configurations are presented in Table S7 (Supporting Information File 1). The computed Ti–O bonds to the organic molecules in the anatase TiO_2_ and rutile TiO_2_ surfaces are lengthen from 0.05 Å to 0.25 Å when EG and GL change their configuration from upright to lying flat.

In summary, DFT calculations show that the chemistry of the organic molecules will depend on the inorganic precursor used and on the surface onto which the films are deposited. We see that EG and GL prefer to lie flat when combined with Ti(DMA)_4_ on the anatase TiO_2_, rutile TiO_2_ and Al_2_O_3_ surfaces and with TiCl_4_ in a anatase TiO_2_ surface but they do not prefer to lie flat when combined with TiCl_4_ on a rutile TiO_2_ surface. The interactions with TiCl_4_ precursor are also less favourable than with Ti(DMA)_4_, partially explained by the strong Ti–Cl bond compared to Ti–N.

## Conclusion

In this study, we used first principles density functional theory (DFT) to investigate the atomistic mechanism of the growth of titanium containing hybrid films known as “titanicones” deposited by MLD. We investigated in detail the chemistry of the MLD process between the TiCl_4_ or Ti(DMA)_4_ inorganic precursors and EG or GL organic molecules. We used anatase TiO_2_, rutile TiO_2_ and Al_2_O_3_ surface models.

Through DFT we calculated the reactivity of TiCl_4_ or Ti(DMA)_4_ inorganic precursors towards the selected surfaces and towards EG and GL organic molecules and predicted the preferred orientation of these organic molecules when combined with TiCl_4_ and Ti(DMA)_4_ in a anatase TiO_2_, rutile TiO_2_ and Al_2_O_3_ surface.

Calculated energetics show that while TiCl_4_ interacts and binds favourably with the anatase TiO_2_ and rutile TiO_2_ surfaces via Ti–O bonds and release of HCl, the interaction with the Al_2_O_3_ surface is not favourable. Ligand loss reactions of TiCl_4_ on the anatase TiO_2_ and rutile TiO_2_ surface are favourable, although there is a notable energy cost for losing the second Cl ligand from surface bound TiCl_3_. Double reactions of EG and GL molecules with TiCl_3_ species are also closely explored. DFT findings show that these molecules can bind with TiCl_3_ species in the anatase TiO_2_ surface in the upright configuration with one terminal OH group and in the flat configuration with two terminal OH groups. In the rutile TiO_2_ surface on the other hand, the preferred orientation of EG and GL molecules is upright. Therefore, we consider the rutile TiO_2_ surface as a suitable surface for the growth of TiCl_4_–EG and TiCl_4_–GL titanicone films where the desired GPCs could be achieved.

DFT calculations show that Ti(DMA)_4_ also interacts and binds favourably with the anatase TiO_2_, rutile TiO_2_ and the Al_2_O_3_ surface via Ti–O bonds. The ligand loss reactions of Ti(DMA)_4_ are associated with the release of H–DMA (H–N(CH_3_)_2_) by-products. A higher reactivity of Ti(DMA)_4_ compared to TiCl_4_ was calculated and this is most probably due to the stronger Ti–Cl bonds present in TiCl_4_ compared to Ti–N bonds present in Ti(DMA)_4_. We show that EG and GL bind favourably with Ti species of Ti(DMA)_4_ via Ti–O bonds and release of H–DMA by-products. However, reaction energetics indicate that these molecules can lie flat and create the unwanted double reactions through the reaction of the two terminal OH groups with the surface fragments. For EG this phenomenon removes active OH groups from the surface and the growth will be less favourable while for GL the third OH group is available and growth proceeds. This analysis supports experimental data on Ti(DMA)_4_–EG and Ti(DMA)_4_–GL film growth and clarifies why for the Ti(DMA)_4_–EG process, the growth stops after 5–10 cycles while for the Ti(DMA)_4_–GL process, the growth proceeds [33].

Temperature is a very important factor for the successful deposition of MLD films. It significantly affects the chemistry between the MLD precursors and especially the behaviour of the organic molecules in the hybrid films. Therefore, we consider this aspect very interesting and worthy of investigating in future work.

## Supporting Information

File 1 Additional data on the geometry of the structures.
