# Supplementary material for: Role of titanium and organic precursors in molecular layer deposition of “titanicone” hybrid materials
Source: Beilstein J Nanotechnol. 2022 Nov 2;13:1240–55. doi: 10.3762/bjnano.13.103 (PMC9644066; doi:10.3762/bjnano.13.103)
Supplement: File 1 — Additional data on the geometry of the structures. [file Beilstein_J_Nanotechnol-13-1240-s001.pdf]

BEILSTEIN JOURNAL OF NANOTECHNOLOGY

# Supporting Information

for

# Role of titanium and organic precursors in molecular layer deposition of “titanicone” hybrid materials

Arbresha Muriqi and Michael Nolan

*Beilstein J. Nanotechnol.* **2022,** *13,* 1240–1255. doi:10.3762/bjnano.13.103

## Additional data on the geometry of the structures

License and Terms: This is a supporting information file under the terms of the Creative Commons Attribution License (https://creativecommons.org/licenses/by/4.0). Please note that the reuse, redistribution and reproduction in particular requires that the author(s) and source are credited and that individual graphics may be subject to special legal provisions.
The license is subject to the *Beilstein Journal of Nanotechnology* terms and conditions: (https://www.beilstein-journals.org/bjnano/terms)

**Table S1:** Computed T–O distances between $TiCl_4$ and the anatase/rutile $TiO_2$ surface

| Structure | Ti-O distance (Å) | Structure | Ti-O distance (Å) |
|---|---|---|---|
| A–$TiCl_3$ | 1.72 | R–$TiCl_3$ | 1.74 |
| A–$TiCl_2$ | 1.78, 1.78 | R–$TiCl_2$ | 1.78, 1.81 |

**Table S2:** Computed Ti–O distances between EG and GL and the $TiCl_3$/$TiCl_2$-terminated anatase/ rutile $TiO_2$ surface and $TiCl_3$/$TiCl_2$ species with surface oxygens after the introduction of EG and GL

| Structure | Ti–O (of EG, GL) distance (Å) | Ti–O (of surface) distance (Å) | Structure | Ti–O (of EG, GL) distance (Å) | Ti–O (of surface) distance (Å) |
|---|---|---|---|---|---|
| A–$TiCl_3$–EG | 1.82 | 1.75 | R–$TiCl_3$–EG | 1.80 | 1.76 |
| A–$TiCl_3$–GL | 1.80 | 1.75 | R–$TiCl_3$–GL | 1.80 | 1.77 |
| A–$TiCl_2$–EG | 1.84 | 1.80, 1.81 | R–$TiCl_2$–EG | 1.82 | 1.82, 1.82 |
| A–$TiCl_2$–GL | 1.82 | 1.81, 1.81 | R–$TiCl_2$–GL | 1.82 | 1.83, 1.82 |

**Table S3:** Computed Ti–O distances between EG and GL in the upright and flat laying configuration and the $TiCl_3$-terminated anatase/rutile $TiO_2$ surface

| Structure | Ti–O distance (Å) | Structure | Ti–O distance (Å) |
|---|---|---|---|
| A–$TiCl_3$–EG–up | 1.80 | R–$TiCl_3$–EG–up | 1.81 |
| A–$TiCl_3$–EG–flat | 1.81 | R–$TiCl_3$–EG–flat | 1.84 |
| A–$TiCl_3$–GL–up | 1.80 | R–$TiCl_3$–GL–up | 1.82 |
| A–$TiCl_3$–GL–flat | 1.81 | R–$TiCl_3$–GL–flat | 1.88 |

**Table S4:** Computed Ti–O distances between $Ti(DMA)_4$ and the anatase/rutile $TiO_2$ and $Al_2O_3$ surfaces

| Structure | Ti–O distance (Å) | Structure | Ti–O distance (Å) | Structure | Ti–O distance (Å) |
|---|---|---|---|---|---|
| A–$(DMA)_3$ | 1.91 | R–$(DMA)_3$ | 1.79 | $Al_2O_3$–$(DMA)_3$ | 1.73 |
| A–$(DMA)_2$ | 1.91, 1.91 | R–$(DMA)_2$ | 1.82, 1.85 | $Al_2O_3$–$(DMA)_2$ | 1.79, 1.80 |
| A–DMA | 1.91, 1.98. 2.03 | R–DMA | 1.86, 1.86, 2.27 | $Al_2O_3$–DMA | 1.83, 1.83, 1.8 |

**Table S5:** Computed Ti–O distances between EG and GL with the TiDMA-terminated anatase/rutile $TiO_2$ and $Al_2O_3$ surfaces

| Structure | Ti–O distance (Å) | Structure | Ti–O distance (Å) | Structure | Ti–O distance (Å) |
|---|---|---|---|---|---|
| A–DMA–EG | 1.80 | R–DMA–EG | 1.80 | $Al_2O_3$–DMA–EG | 1.86 |
| A–DMA–GL | 1.79 | R–DMA–EG | 1.80 | $Al_2O_3$–DMA–EG | 1.87 |

**Table S6:** Computed Ti–O distances between TDMAT and the anatase/rutile $TiO_2$ and $Al_2O_3$ surfaces after the introduction of EG and GL

| Structure | Ti–O distance (Å) | Structure | Ti–O distance (Å) | Structure | Ti–O distance (Å) |
|---|---|---|---|---|---|
| A–DMA–EG | 1.90, 1.98, 2.0 | R–DMA–EG | 1.86, 1.87, 2.23 | $Al_2O_3$–DMA–EG | 1.84, 1.85, 1.85 |
| A–DMA–GL | 1.91, 1.98, 2.0 | R–DMA–EG | 1.86, 1.87, 2.21 | $Al_2O_3$–DMA–EG | 1.83, 1.85, 1.85 |

**Table S7:** Computed Ti–O distances between EG and GL in the upright and flat laying configuration and the TiDMA-terminated anatase/rutile $TiO_2$ and $Al_2O_3$ surfaces

| Structure | Ti–O distance (Å) | Structure | Ti–O distance (Å) | Structure | Ti–O distance (Å) |
|---|---|---|---|---|---|
| A–DMA–EG–up | 1.80 | R–DMA–EG–up | 1.79 | $Al_2O_3$–DMA –EG–up | 1.87 |
| A–DMA–EG–flat | 1.88 | R–DMA–EG–flat | 1.85 | $Al_2O_3$–DMA –EG–flat | 2.08 |
| A–DMA–GL–up | 1.79 | R–DMA–GL–up | 1.79 | $Al_2O_3$–DMA –GL–up | 1.86 |
| A–DMA–GL lat | 1.86 | R–DMA–GL–flat | 1.84 | $Al_2O_3$–DMA –GL–flat | 2.11 |
